# Effect of 2D and 3D Culture Microenvironments on Mesenchymal Stem Cell-Derived Extracellular Vesicles Potencies

**DOI:** 10.3389/fcell.2022.819726

**Published:** 2022-02-14

**Authors:** Gina D. Kusuma, Anqi Li, Dandan Zhu, Hannah McDonald, Ishmael M. Inocencio, Daniel C. Chambers, Kenneth Sinclair, Haoyun Fang, David W. Greening, Jessica E. Frith, Rebecca Lim

**Affiliations:** ^1^ The Ritchie Centre, Hudson Institute of Medical Research, Melbourne, VIC, Australia; ^2^ Department of Obstetrics and Gynaecology, Monash University, Melbourne, VIC, Australia; ^3^ Queensland Lung Transplant Service, The Prince Charles Hospital, Brisbane, QLD, Australia; ^4^ School of Clinical Medicine, Faculty of Health Sciences, University of Queensland, Brisbane, QLD, Australia; ^5^ Molecular Proteomics, Baker Heart and Diabetes Institute, Melbourne, VIC, Australia; ^6^ Baker Department of Cardiometabolic Health, The University of Melbourne, Melbourne, VIC, Australia; ^7^ Baker Department of Cardiovascular Research, Translation and Implementation, La Trobe University, Melbourne, VIC, Australia; ^8^ Central Clinical School, Monash University, Melbourne, VIC, Australia; ^9^ Department of Materials Science and Engineering, Monash University, Melbourne, VIC, Australia

**Keywords:** mesenchymal stem cells, extracellular vesicle, spheroids, microenvironment, lung fibrosis, inflammation, immunomodulation, 3D culture

## Abstract

Therapeutic benefits of mesenchymal stem cells (MSCs) are now widely believed to come from their paracrine signalling, i.e. secreted factors such as cytokines, chemokines, and extracellular vesicles (EVs). Cell-free therapy using EVs is an active and emerging field in regenerative medicine. Typical 2D cultures on tissue culture plastic is far removed from the physiological environment of MSCs. The application of 3D cell culture allows MSCs to adapt to their cellular environment which, in turn, influences their paracrine signalling activity. In this study we evaluated the impact of 3D MSCs culture on EVs secretion, cargo proteome composition, and functional assessment in immunomodulatory, anti-inflammatory and anti-fibrotic properties.

MSC-EVs from 2D and 3D cultures expressed classical EV markers CD81, CD63, and CD9 with particle diameter of <100 nm. There were distinct changes in immunomodulatory potencies where 3D cultures exhibited reduced indoleamine 2,3-dioxygenase (IDO) activity and significantly reduced macrophage phagocytosis. Administration of 2D and 3D EVs following double dose bleomycin challenge in aged mice showed a marked increase of bodyweight loss in 3D group throughout days 7–28. Histopathological observations of lung tissues in 3D group showed increased collagen deposition, myofibroblast differentiation and leukocytes infiltrations. Assessment of lung mechanics showed 3D group did not improve lung function and instead exhibited increased resistance and tissue damping. Proteome profiling of MSC-EV composition revealed molecular enrichment of EV markers (compared to parental cells) and differential proteome between EVs from 2D and 3D culture condition associated with immune-based and fibrosis/extracellular matrix/membrane organization associated function.

This study provides insight into distinct variation in EV protein composition dependent on the cellular microenvironment of the parental cells, which could have implications in their therapeutic effect and potency. Overall, this work suggests that EVs produced from 3D MSC cultures did not enhance typical MSC-EV properties expected from 2D cultures (immunomodulation, anti-fibrotic, anti-inflammatory). The outcome highlights critical differences between MSC-EVs obtained from different culture microenvironments, which should be considered when scaling up MSC culture for clinical manufacturing.

## Introduction

Mesenchymal stem cells (MSCs) are excellent candidates for a range of therapeutic applications (Levy et al., 2020). Much of the clinical interest in MSCs is due to their known anti-inflammatory and anti-fibrotic properties. These are mediated by the secretion of various cytokines and soluble factors, which in turn modulate immune cell activity and promote tissue generation. More recently, MSC-derived extracellular vesicles (EVs) have also attracted attention as therapeutic products produced by MSCs which may have potential use in tissue repair and regeneration (Baglio et al., 2012; György et al., 2015; Chen et al., 2017; Claridge et al., 2021a).

The biological functions of EVs and their components vary according to their cellular origins. The therapeutic capacity of MSC-EVs derived from different tissues have been tested in various disease models, demonstrating a similar or even superior functional potential to MSCs themselves (Burrello et al., 2016; Riazifar et al., 2017). The EVs cargo content typically reflect their parental cells, cell culture conditions and microenvironmental stimuli that triggered their release. The effects of MSC-derived EVs can be enhanced under specific physical and biological stimuli such as hypoxia, LPS, IL-6, IFN-γ, TNF-α, IL-1β, which not only increase EV production but may also alter their components, leading to an enhancement of beneficial effects (Lamichhane et al., 2014; Phan et al., 2018; Kwon et al., 2021). Furthermore, MSCs may lodge and initially obstruct small vessels in organs such as lungs (Wang et al., 2014; Levy et al., 2020). EVs, in contrast, have no vascular obstructive effect or apparent adverse effects. These properties suggest that EVs could be safely and easily used in therapies such as for respiratory diseases (Abreu et al., 2016; Fujita et al., 2018; Claridge et al., 2021a).

MSCs are commonly cultured as monolayers using conventional tissue culture plastic (TCP). Pre-conditioning MSCs under physiological conditions, for example: hypoxia, 3D environments, matrix stiffness and many others have been shown to optimise their therapeutic efficacy (Kusuma et al., 2017). There has been increasing interest in culturing MSCs as multicellular spheroids in which the cells interact with each other but do not adhere to any external surface (Egger et al., 2018). Such MSC spheroids can be cultured statically, for example, in low-attachment plates but are also compatible with suspension culture in bioreactors, providing the potential to be readily scaled up to produce the large cell numbers required for therapeutic application (Frith et al., 2012). Compared to MSCs cultured as monolayers on TCP, MSCs from spheroid cultures have lower stiffness, altered expression of cell surface antigens and an increased ability to differentiate along the osteogenic and adipogenic lineages (Frith et al., 2010; Tietze et al., 2019). CXCR4 (involved in MSC homing in the body) and IL-24 (anti-cancer factor) expressions have shown to be increased in MSC spheroids (Frith et al., 2010). When cultivated as 3D aggregates or spheroids, MSCs display increased angiogenic, anti-inflammatory, and immunomodulatory effects as well as improved stemness and survival rates after transplantation (Xie et al., 2021). It has been suggested that cultivation of MSC spheroids could modulate their differentiation, proliferation and paracrine effects (Egger et al., 2018). However, while appearing beneficial in several settings, the impact of 3D spheroid culture on MSCs secretory production, specifically EV cargo and yield, has not been determined. This is critical both for suppression of inflammation and, in allogeneic uses, immune evasion.

One promising application of MSCs is to treat respiratory diseases where MSCs have been characterised extensively and tested in various Phase 1-3 clinical trials for conditions such as idiopathic pulmonary fibrosis (IPF), acute respiratory distress syndrome, chronic obstructive pulmonary disease, silicosis, and many others (Toonkel et al., 2013; Stabler et al., 2015; Averyanov et al., 2020). IPF is a chronic, progressive and irreversible respiratory disease characterized by diffuse alveolar epithelial cell injury and structural remodelling (Toonkel et al., 2013). Massive deposition of heterogeneously distributed extracellular matrix (ECM) components in the alveolar parenchyma is a major characteristic of IPF (Herrera et al., 2018). There is typically no response to general anti-inflammatory therapies such as glucocorticosteroids and immunosuppressants. Lung transplantation is currently the most effective therapy, with a 50% chance survival rate 5 years post-transplantation (Chambers et al., 2021). Drugs such as pirfenidone and nintedanib have been used for patients who are ineligible for transplantation in an attempt to slow the progression of disease. These drugs target the progression of fibrosis but neither are curative (Azuma et al., 2005; Dinh et al., 2020). Therefore, new treatment strategies such as cell-free therapies have emerged to utilise the regenerative ability of stem cells but without the high cost of goods, safety and logistical challenges.

To mimic IPF progression, we chose to induce pulmonary fibrosis in mice using bleomycin challenge as this approach is the most widely used agent and well-characterised model (Della Latta et al., 2015; Srour and Thebaud 2015; Miles et al., 2020). Intratracheal and intranasal administration of bleomycin can induce damage to alveolar epithelial cells, increase the presence of alveolar inflammatory cells, fibroblast proliferation, and synthesis of ECM (Della Latta et al., 2015). Aged mice (∼12 months) were used in combination with double administration of bleomycin where both measures were implemented to resemble fibrotic phenotype and pathological changes (Dutta and Sengupta 2016). Given the disease heterogeneity, it is important that the bleomycin model could accurately reflect pathogenic mechanisms in order to accelerate the translation of research findings from the model to the appropriate patient population.

Therefore, this study investigated the therapeutic potential for 2D and 3D MSC-EVs in the setting of lung fibrosis to enable direct comparison to the existing body of knowledge in regard to the safety profile, immunosuppressive, anti-inflammatory, and anti-fibrotic effects of MSC-EVs. We evaluated the impact of 2D and 3D spheroids culture on the secretion and proteome composition of EVs released by MSCs. We aimed to compare the biological implications of MSC-EV production in 2D and 3D culture systems and investigate their immunomodulatory, anti-inflammatory and anti-fibrotic properties both *in vitro* and *in vivo*.

## Materials and Methods

### Isolation and Purification of Extracellular Vesicles

Bone marrow MSCs (*n* = 6 healthy donors, Lonza Biosciences) were maintained and passaged on tissue culture plastic using DMEM/LG with 10% FBS. MSCs were aggregated into 3D spheroids (cell density = 2.5 × 10^4^/spheroid) using non-adherent 96-well plates and cultured with DMEM/0.5% FBS/0.25% methylcellulose for 24 h at 37°C and 5% CO_2_. Both 2D and 3D MSCs were cultured at a density of 5 × 10^4^ cells/mL using Ultraculture (Lonza Biosciences) with 1% Glutamax (Thermo Fisher) serum free media formulation for 4 days at 37°C and 5% CO_2_. Conditioned media from MSC 2D and 3D cultures were then processed for EVs isolation by serial ultracentrifugation (Théry et al., 2006; Rai et al., 2021). CM was centrifuged at 300 *g* for 10 min at 4°C, at 2000 *g* for 10 min at 4°C, and at 10,000 *g* for 30 min at 4°C (VX22N high speed centrifuge equipped with R20A2 rotor, VWR, United States) to remove cells, dead cells, and cellular debris respectively. The supernatant was subjected to ultracentrifugation at 100,000 g for 120 min at 4°C (V100X ultracentrifuge equipped with P28S rotor, VWR, United States). The pellets were washed in PBS and subjected to ultracentrifugation at 100,000 *g* for 70 min at 4°C. The pellets containing EVs were resuspended in PBS prior to storage at −80°C. Protein yield was determined by measuring absorbance at 562 nm using the Pierce BCA protein assay kit (Thermo Fisher).

### 3D Spheroid Evaluation

Physical characteristics of the spheroids at day 4 of culture were evaluated by measuring spheroid area, circularity, perimeter, and Feret’s diameter using Image J software. These parameters were measured from 3 different MSC donors with at least 20 spheroids included per donor and each individual value was shown in the scatter plot.

### Transmission Electron Microscopy

EV samples were prepared for transmission electron microscopy on formvar carbon-coated 400-mesh copper grids (Agar Scientific, Stansted, United Kingdom) which were treated with plasma to render the carbon surface hydrophilic. EVs (3 μL) were placed onto a carbon grid and incubated for 20 min for adsorption, with the grids rinsed 3 times in Sorenson’s buffer and fixed with 1% (w/v) glutaraldehyde for 5 min. Again, the grids were washed; 8 times in ultrapure water. The samples were stained using 2% (w/v) uranyl acetate (UA) for 5 min after which 0.5 µL final embedding solution (ES; 700 µL of 2% (w/v) methyl cellulose, 100 µL 3% UA, 187 µL Milli-Q water, 12.5 µL of 2% Phosphotungstic acid) was applied. Excess ES was removed, and the grids were air-dried for 15 min. The samples were analysed using a Tecnai 12 Transmission Electron Microscope, operating at an accelerating voltage of 120 kV. Images were acquired using a Megaview III CCD camera and AnalySIS camera control software (Olympus).

### Extracellular Vesicles Particle Quantification and Phenotyping

The particle size, quantity and surface protein characteristics of EVs were analysed through single particle interferometric reflectance imaging sensing (SP-IRIS) using the ExoView platform (Nanoview Biosciences, United States). SP-IRIS uses a multiplex microarray chip for the immune-capture of commonly expressed EV tetraspanin proteins CD9, CD63, CD81 (ExoView Human Tetraspanin kit, EV-TETRA-C). SP-IRIS analyses EVs using visible light interference for size measurements and fluorescence for protein profiling. EV was diluted with PBS containing 0.05% Tween 20. Up to 35 µL of this sample was carefully pipetted into the silicon chip coated with individual antibody spots against human CD9, CD63, CD81 as well as mouse IgG as isotype controls. Following overnight incubation in a 24-well plate, chips were washed three times on an orbital shaker with Solution A. Then, the chips were incubated for 1 h at RT with CD9-AF488, CD81-AF555, CD63-AF647 (0.5 μg/ml). Chips were washed once in Solution A, three times in Solution B, and once in deionized water. Chips were carefully removed from the 24-well plate, washed further in deionized water and removed for drying. Image and data acquisition for each chip was performed with the ExoView R100 reader using the nScan 2.8.10 acquisition software (NanoView Biosciences). Data analysis was performed with sizing thresholds set to 50–200 nm diameter and analysed using NanoViewer 2.8.10 and ExoViewer 3 (NanoView Biosciences).

### Indoleamine 2,3-Dioxygenase Activity Assay

The use of IFN-γ priming is widely accepted as a surrogate potency assay for the immunomodulatory response of MSCs (Chinnadurai et al., 2018). 2D and 3D MSCs were cultured in Ultraculture media for 4 days with and without IFN-γ (2.5 ng/ml). Cells were plated at a cell seeding density of 1 × 10^5^ cells/well for 2D MSCs or the equivalent of four spheroids (2.5 × 10^4^ cells/spheroids) for 3D MSCs in a 6-well plate. CM were collected and assayed for IDO activity in duplicates. IDO activity was measured by quantifying the concentration of kynurenine, the product of the enzymatic activity of IDO (François et al., 2012; Chinnadurai et al., 2018).

### Macrophage Phagocytosis

Immortalised murine bone marrow derived macrophages (iBMDMs) (a gift from A/Prof Ashley Mansell, Hudson Institute of Medical Research, Melbourne, Australia) were plated in 96-well black flat-bottom plates at a density of 1 × 10^5^ cells per well, where each well contained 100 μL of DMEM-F12 + 10% FBS (Rosli et al., 2019). Plates were then incubated at 37°C/5% CO_2_ for overnight to allow for cell attachment. Media from each well was replaced with fresh media and treatments (5 μg/mL EVs and controls) were added. Following 24-hour treatment, the culture medium was replaced with 100 μL of 100 μg/ml pHrodo solution (pHrodo Red E. coli Bioparticles Conjugate, P35361, ThermoFisher Scientific) in uptake buffer (HBSS +5% (v/v) FBS). Following 1 h incubation at 37°C, pHrodo solution was replaced with 100 μL of uptake buffer prior to plate reading using a fluorescence plate reader (Molecular Devices, SpectraMax i3).

### IL-2 Production by Jurkat Cells

Jurkat cells (Clone E6-1, cat#TIB-152, ATCC) were cultured at 1 × 10^6^/ml in 96-well plate in RPMI supplemented with 10% FBS and 1% Antibiotic-Antimycotic. Cells were stimulated Dynabeads Human T-activator CD3/CD28 (11161D, Thermo Fisher) at 1:1 cell to bead ratio for 24 h. EV samples were added at 5 μg/ml and control samples are Jurkat cells without any EV. Supernatant was collected and assayed for IL-2 production in duplicate using ELISA kit (#88–7025–88, Thermo Fisher) following manufacturer instructions. Briefly, a high binding ELISA plate was coated with capturing antibody overnight at 4°C. The ELISA plate was blocked with diluting buffer for 1 hour before incubation with sample at RT for 2 h in with agitation. Followed with incubation, the sample was incubated with detecting antibody and developed with 3,3′,5,5′-Tetramethylbenzidine (TMB) substrate. The reaction was stopped after 15 min using 2N sulphuric acid. The IL-2 concentration was determined by absorbance at 450 nm using a microplate reader (Molecular Devices, SpectraMax i3).

### *In vitro* Lung Fibroblast Activation Assay

Human lung fibroblasts (HLF) (CCL 171, ATCC) were plated at a density of 3 × 10^4^ cells/cm^2^ in a 96-well plate in DMEM supplemented with 1% Glutamax and 0.5% FBS. Following 24 h of incubation at 37°C, 5% CO_2_ and serum starvation, the media is replaced with fresh medium containing 5 ng/ml TGF-β1 (100–21, Peprotech, United States) per well. After 24-hour incubation at 37°C and 5% CO_2_, 5 μg/ml of MSC-EV samples was added to each well. Samples were then collected after 24 h and centrifuged at 3000 *g* for 5 min. Collagen content in the conditioned media from HLF cultures was measured using Sircol collagen assay (Biocolor).

### Patient-Derived Lung Mesenchymal Stem Cells Proliferation Assay

Human lung tissue-derived MSCs (LT-MSCs) were isolated from digested parenchymal lung tissue as previously described (Sinclair et al., 2016b). This study was approved by the Human Research Ethics Committee, Metro North Hospital and Health Services, The Prince Charles Hospital (HREC/13/QPCH/96). Lung MSCs were isolated from healthy non-smoking patients undergoing a lung resection for recurrent pneumothorax. IPF lung MSCs were isolated from the lower lobe of patients diagnosed with IPF. Cells were cultured and passaged up to P5 in DMEM-Low glucose with 10% FBS and 1% antibiotic-antimycotic. To assess changes in cell proliferation, LT-MSCs were plated in a 96-well plate at a cell seeding density of 1 × 10^4^ cells/cm^2^ and 5 μg/ml of 2D and 3D MSC-EVs were added in triplicates. Cells were incubated for up to 3 days and MTS assay was performed according to the manufacturer’s instructions. At each time point, 20 µL of CellTiter 96^®^ AQueous One Solution Cell Proliferation Assay reagent (Promega, United States) were added into each well and incubated for 2 h at 37°C. The absorbance was assessed at 490 nm using a microplate reader (Molecular Devices, SpectraMax i3).

### *In vivo* Lung Fibrosis Model

Animal experiments were carried out with approval by Monash University Animal Ethics Committee (MMCA 2015/36) and conducted in accordance with the Australian Code of Practice for the Care and Use of Animals for Scientific Purposes. Female C57Bl/6 mice aged 48–52 weeks were housed in pathogen free Monash Medical Centre Animal facility with ad libitum access to food and water. Animals were divided into 5 groups (*n* = 6–8/group) and, other than the healthy untreated (saline) group, every animal received bleomycin intranasally. Mice were put under using 2.5% isoflurane inhalation at 0.4 L/min and were given intranasal administration of 0.4 units of bleomycin (0.01 units/g, Pro Pharmaceuticals Group, Lot: 1805002) in a total volume of 30 μL at days 1 and 7. This dose was shown to be optimal to induce pulmonary fibrosis without animal mortality as determined by preliminary dose titration study (data not shown). Animals in the saline group received an intranasal administration of 30 µL saline alone.

Animals were then treated with intranasal administration of EVs or pirfenidone 24 h after the 2^nd^ bleomycin challenge at day 8. Each animal was randomly allocated to receive either 10 μg EV (from 6 pooled MSC donors), pirfenidone (125 mg/kg), or saline alone via intranasal administration. Animal welfare including feeding, weight, general well-being and clinical score was monitored and charted daily. Mice were subjected to lung function testing at 28 days followed directly by lung tissues collection for histology and protein analysis. Lung tissues were collected as previously described (Tan et al., 2018).

### Lung Function Testing

Invasive lung function measurements were taken at day 28. Briefly, mice were anaesthetized using a combination of ketamine/xylazine (0.1 ml/10 g bodyweight) and tracheotomized using an 18G cannula, which was connected to the Y-tubing of the plethysmograph. Animals were mechanically ventilated using a computer-controlled piston ventilator and lung mechanics was measured using FlexiVent (Scireq, Canada). The linear single-frequency forced oscillation technique was used to assess total respiratory system resistance (Rrs) with the Snapshot-150 perturbation at a respiratory rate of 150 breath/min, tidal volume of 10 ml/kg, and 3 cmH_2_O positive end-expiratory pressure. The broadband forced oscillation technique was used to determine tissue damping (*G*) with the Quicktime-3 perturbation. Volume-driven P-V loops were formed from incrementally inflating the lungs to 30 cm H_2_O from functional residual capacity, which was defined as 3 cm H_2_O. Area of P-V curve was calculate by the Flexivent software and the data was used to assess hysteresis. All measurements of lung mechanics were conducted in mice with intact chest walls.

### Histology

After performing lung function measurements, mice were anaesthetised through CO_2_ asphyxiation and exsanguinated. The heart was perfused using 20 ml of saline through the left ventricle. The trachea was cannulated and the right lung tied off. The left lung was inflated with 4% paraformaldehyde (PFA), tied off and collected for histological analysis. The right lung was collected and snap frozen on dry ice for protein analysis. PFA-fixed lungs were embedded in paraffin wax and sectioned at 5 µm thickness. Sections were deparaffinised, rehydrated and stained with haematoxylin and eosin to identify various cell types present. To visualise and quantify collagen deposition, Sirius Red staining was performed using 0.1% w/v Sirius Red F3B (Sigma Aldrich). Sirius Red stained sections were scanned at ×20 magnification using the Aperio Scanscope AT Turbo. Ten representative, non-overlapping regions were randomly selected from each section and collagen content was quantified using ImageJ software (NIH). The percentage of tissue that stained positive for collagen was calculated by: (% area of positive collagen staining/% area of tissue) × 100. The % of collagen stained tissue calculated from each region was averaged and the mean % of collagen stained tissue of animals in each group, was statistically analyzed between the control and experimental groups.

### Immunofluorescence Staining

Tissue sections were deparaffinised, rehydrated and subjected to heat-mediated antigen retrieval using sodium citrate buffer (10 nM, pH 6.0) for 10 min. Sections to be immunostained for alpha-Smooth muscle actin (α-SMA) were further permeabilized with 0.1% Triton-X-100 for 15 min at room temperature (RT). All sections were blocked with serum-free protein block (Dako, Agilent) for 30 min, prior to adding any primary antibodies for 4°C, overnight incubation. CD45 (1:100, ab10558, Abcam) and *α*-SMA (1:50, ab5694, Abcam) antibodies were then applied to prepared sections. Proteinase K antigen retrieval (20 μL/ml) was performed for 3 min at RT to tissue sections to be immunostained for F4/80, before the primary antibody was applied (1:100, MCA497G, Serotec). Secondary antibodies applied were anti-rabbit 568 (A-10042, Invitrogen) for CD45 (1:500) and *α*-SMA (1:100), and anti-rat 488 (A-11006, Invitrogen) for F4/80 (1:200) for 1 h incubation at RT. All sections were nuclear stained with DAPI (1:5,000). Sudan Black staining was performed (0.1% (w/v) in 70% (v/v) Ethanol) for 30 min to remove autofluorescence. Fluorescently stained slides were scanned at ×40 magnification using the Olympus VS120 Virtual Slide Microscope. 10 representative non-overlapping regions per section were randomly selected for analysis using ImageJ software (NIH). The mean fluorescence intensity (MFI) of CD45, F4/80 and a-SMA was measured by averaging the Integrated Density measurement of the 10 regions per section. Statistical analysis of the MFI of CD45, F4/80 and *α*-SMA across the control and treatment groups was then performed.

### Proteomics Sample Preparation and Liquid Chromatography-Tandem Mass Spectrometry

Parental cells and derived EVs from 2D and 3D MSCs (*n* = 5) were solubilized in 1% (v/v) sodium dodecyl sulphate (SDS), 50 mM HEPES pH 8.0, and quantified by microBCA protein assay kit (Thermo Fisher Scientific). For mass spectrometry-based proteomics, samples (7.5 μg in 50 μL) were normalized and reduced with 10 mM dithiothreitol (DTT) for 60 min at 25°C followed by alkylation with 20 mM iodoacetamide for 30 min at 25°C in the dark (Claridge et al., 2021b; Kompa et al., 2021). The reaction was quenched to a final concentration of 20 mM DTT. Magnetic beads were prepared by mixing Sera-Mag Speed Beads A and B at 1:1 (v:v) ratio and washing twice with 200 µL MS-water. Magnetic beads were reconstituted to a final concentration of 100 μg/μL. Magnetic beads were added to the samples at 10:1 beads-to-protein ratio and ethanol added for a final concentration of 50% (v/v). Protein-bound magnetics beads were washed three times with 200 µL of 80% ethanol and reconstituted in 50 mM TEAB pH 8 and digested with trypsin (Promega, V5111) at a 1:50 enzyme-to-substrate ratio for 16 h at 37°C at 1,000 rpm. The peptide mixture was acidified to a final concentration of 2% formic acid, and centrifuged at 20,000 g for 1 min. The peptide digests were collected from the supernatant and kept frozen at −80°C and dried by vacuum centrifugation, reconstituted in 0.07% trifluoroacetic acid, and quantified by Fluorometric Peptide Assay (Thermo Scientific) as per manufacturer’s instructions.

Peptides were analysed on a Dionex UltiMate NCS-3500RS nanoUHPLC coupled to a Q-Exactive HF-X hybrid quadrupole-Orbitrap mass spectrometer equipped with nanospray ion source in positive mode as described (Claridge et al., 2021b). Peptides (250 ng) were loaded (Acclaim PepMap100 C18 3 μm beads with 100 Å pore-size, Thermo Fisher Scientific) and separated (1.9-µm particle size C18, 0.075 × 250 mm, Nikkyo Technos Co. Ltd.) with a gradient of 2–28% acetonitrile containing 0.1% formic acid over 95 min followed by 28–80% from 95–98 min at 300 nL min^−1^ at 55°C (butterfly portfolio heater, Phoenix S&T). An MS1 scan was acquired from 350 to 1,650 m/*z* (60,000 resolution, 3 × 10^6^ automatic gain control (AGC), 128 msec injection time) followed by MS/MS data-dependent acquisition (top 25) with collision-induced dissociation and detection in the ion trap (30,000 resolution, 1 × 10^5^ AGC, 60 m sec injection time, 28% normalized collision energy, 1.3 m/*z* quadrupole isolation width). Unassigned precursor ions charge states and slightly charged species were rejected and peptide match disabled. Selected sequenced ions were dynamically excluded for 30 s. Data was acquired using Xcalibur software v4.0 (Thermo Fisher Scientific).

### Database Searching and Protein Identification

Identification and quantification of peptides was performed using MaxQuant (v1.6.14.0) and Andromeda as previously described (Cox et al., 2011; Tyanova et al., 2016; Evans et al., 2020). Tandem mass spectra were searched as a single batch against the *Homo sapiens* reference proteome (74,823 entries, downloaded Jan-2020) supplemented with common contaminants. Search parameters were as follows: carbamidomethylated cysteine as fixed modification, oxidation of methionine and N-terminal protein acetylation as variable modifications, trypsin/P as proteolytic enzyme with ≤2 missed cleavage sites, search tolerance 7 ppm, fragment ion mass tolerance 0.5 Da, <1% false discovery rate on peptide spectrum match with target-decoy approach at peptide and protein levels, match between runs selected, and label free quantification (LFQ) algorithm employed.

### Data Analysis and Bioinformatics Pipeline

Protein lists for samples were generated in Perseus (v1.6.14.0) based on ≥ 2 peptides. Principal component analysis was performed in Perseus with missing values imputed from normal distribution (width 0.3, downshift 1.8). Functional/pathway enrichment map analysis was performed using Gene Ontology Cellular Component (GOCC), Gene Ontology Biological Process (GOBP), and Gene Ontology Molecular Function (GOMF). Enrichment terms of EV in comparison to parental cells in each condition was determined by Benjamin-Hochberg, FDR<0.05. Functional enrichment annotations were analyzed using gProfiler (*p* < 0.05). All data points are visualised using R (ggplot2) with combined volcano plot and balloon plot.

### Statistical Analysis

All statistical analyses were performed using GraphPad Prism v9.0 (GraphPad software Inc., United States) and the values were expressed as the mean ± SEM. *In vitro* assays were analyzed by an unpaired *t*-test and one-way ANOVA followed by Tukey’s post hoc test. The *in vivo* data were analyzed by a one-way ANOVA followed by Tukey’s post hoc test for multiple comparisons except for bodyweight loss data which were analyzed by a two-way ANOVA. In each case, differences between means were significant when *p* < 0.05.

## Results

### Characterization of 3D Mesenchymal Stem Cells Spheroid Cultures

**FIGURE 1:**
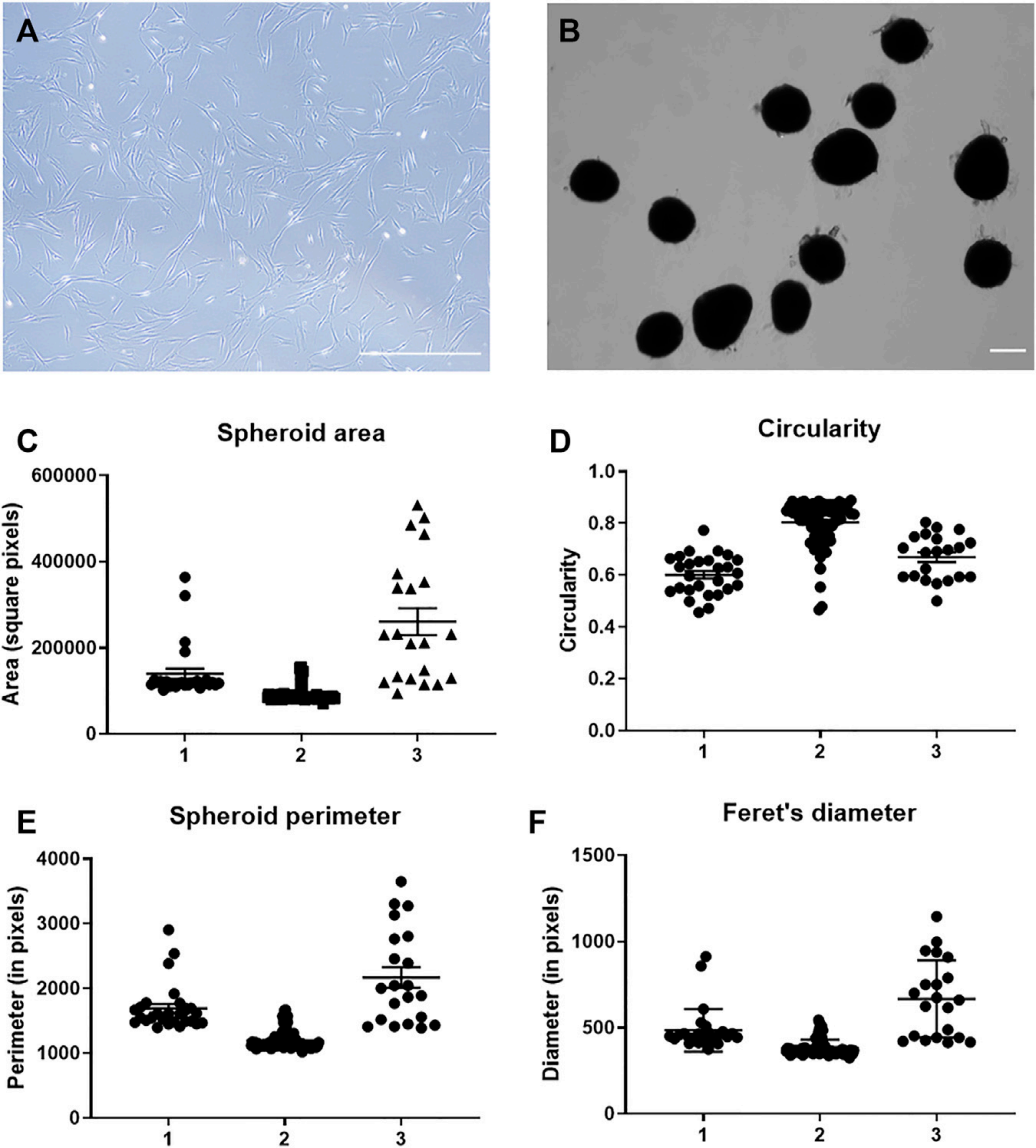
Characterization of MSC spheroids. **(A)** Representative photomicrograph of MSC in 2D culture on tissue culture plastic and **(B)** MSC in 3D spheroid culture at day 4 of culture. Scale bar = 250 µm. Quantitation of **(C)** spheroid area, **(D)** spheroid circularity scales from 0–1 where a value of 1 represents a perfect circle, **(E)** spheroid perimeter and **(F)** Feret’s diameter performed at day 4 of culture with *n* = 3 MSC donors.

2D MSCs were cultured in monolayer using standard TCP for passaging up to P5 (Figure 1A). MSC spheroids with approximately 2.5 × 10^4^ cells were formed via forced aggregation in non-adherent 96-well microplates. After overnight incubation in the microplates, MSCs aggregated into relatively uniform spheroids of distinct sizes (Figure 1B) and were transferred to static suspension culture to prevent surface attachment and agglomeration of individual aggregates. Conditioned media were collected from 3D MSCs after 4 days incubation in Ultraculture serum-free media for downstream EV isolation. Physical characterisation of 3D spheroids was performed at day 4 of culture and these parameters (area, circularity, perimeter, and Feret’s diameter) were presented in Figures 1C–F. This evaluation showed that among the 3 MSC donors these parameters were consistent and these spheroids were of uniform shape and near spherical.

### Characterization of Extracellular Vesicles Produced From 2D and 3D Cultures

**FIGURE 2:**
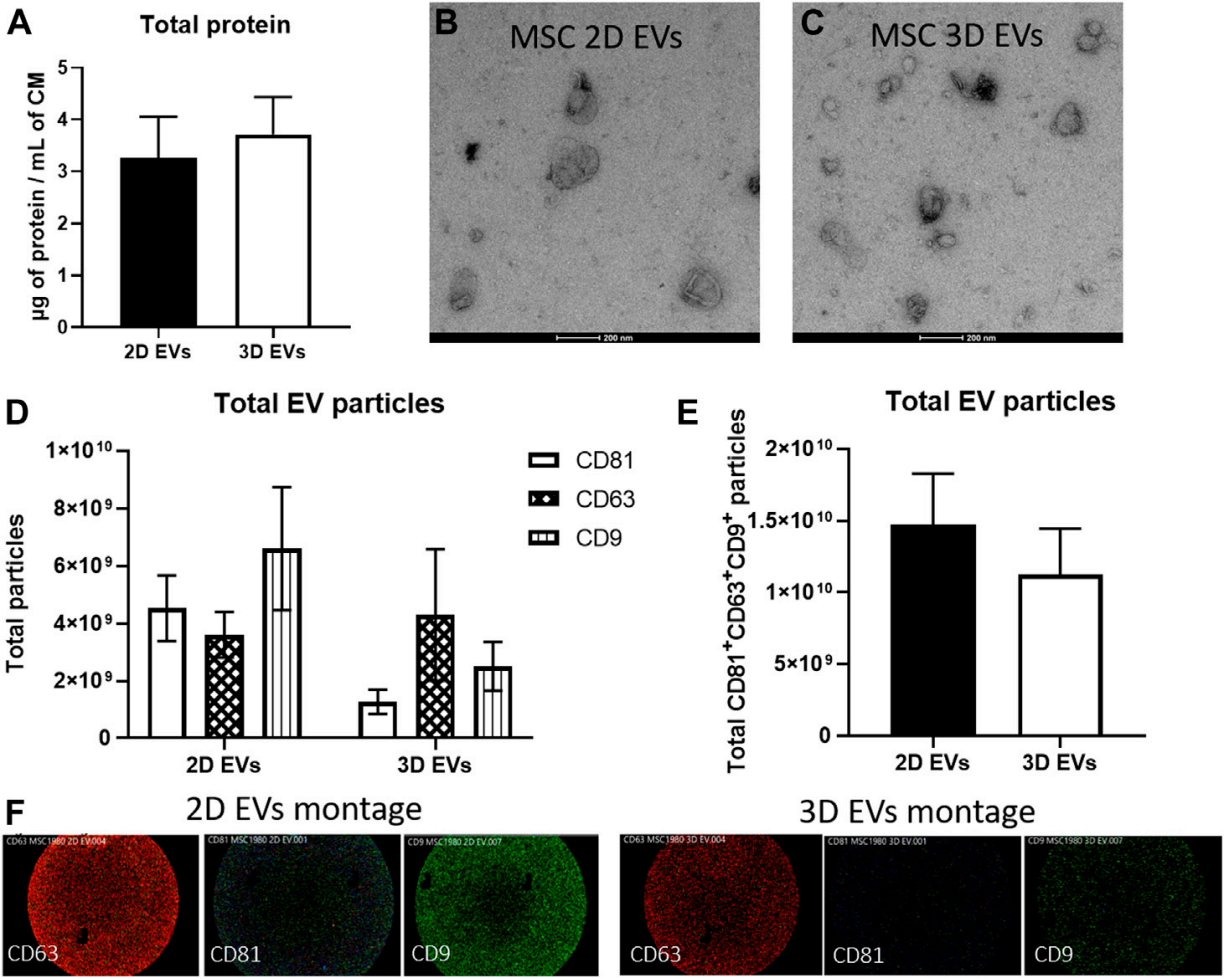
Characterization of EVs produced by MSCs in 2D and 3D spheroid culture. **(A)** BCA assay to determine total protein yield. **(B–C)** Negative staining images of MSC 2D and 3D EVs on transmission electron microscope. Scale bar = 200 µm **(D)** EV particle analysis and phenotyping for individual EV tetraspanin markers CD63, CD81, and CD9 expressions using SP-IRIS. **(E)** Total CD63^+^CD81^+^CD9^+^ particles from each sample, *n* = 3 each. **(F)** Visualisation of multi-colour fluorescence signals obtained from SP-IRIS imaging which represents single vesicle events. Detection performed with CD81-AF555 (green), CD63-AF647 (red), and CD9-AF488 (blue).

EVs were isolated from conditioned media collected from 2D and 3D MSC cultures using serial ultracentrifugation. EVs were quantified using BCA assay to obtain total crude protein yield and both 2D and 3D MSC-EVs did not show any significant difference (Figure 2A). Transmission electron microscopy revealed that both 2D and 3D MSC-EVs exhibited cup-shaped and spherical morphology characteristics of EVs (Figures 2B,C). Quantification of EV particle yield and characteristics showed that EV production and tetraspanin expressions were not affected by 3D culture. SP-IRIS analysis showed expression of classical EV surface markers of CD81^+^ CD63^+^ CD9^+^ (Figures 2D,E) (Théry et al., 2019). 2D and 3D MSC-EVs had comparable total particle counts. Representative co-localisation data and particle size distribution confirmed that both 2D and 3D MSC-EVs have an average particle size of <100 nm, which is the expected range for EVs isolated from cell culture supernatant (Supplementary Figure S1).

### *In vitro* Testing of the Functional Effects of 2D and 3D Extracellular Vesicles

**FIGURE 3:**
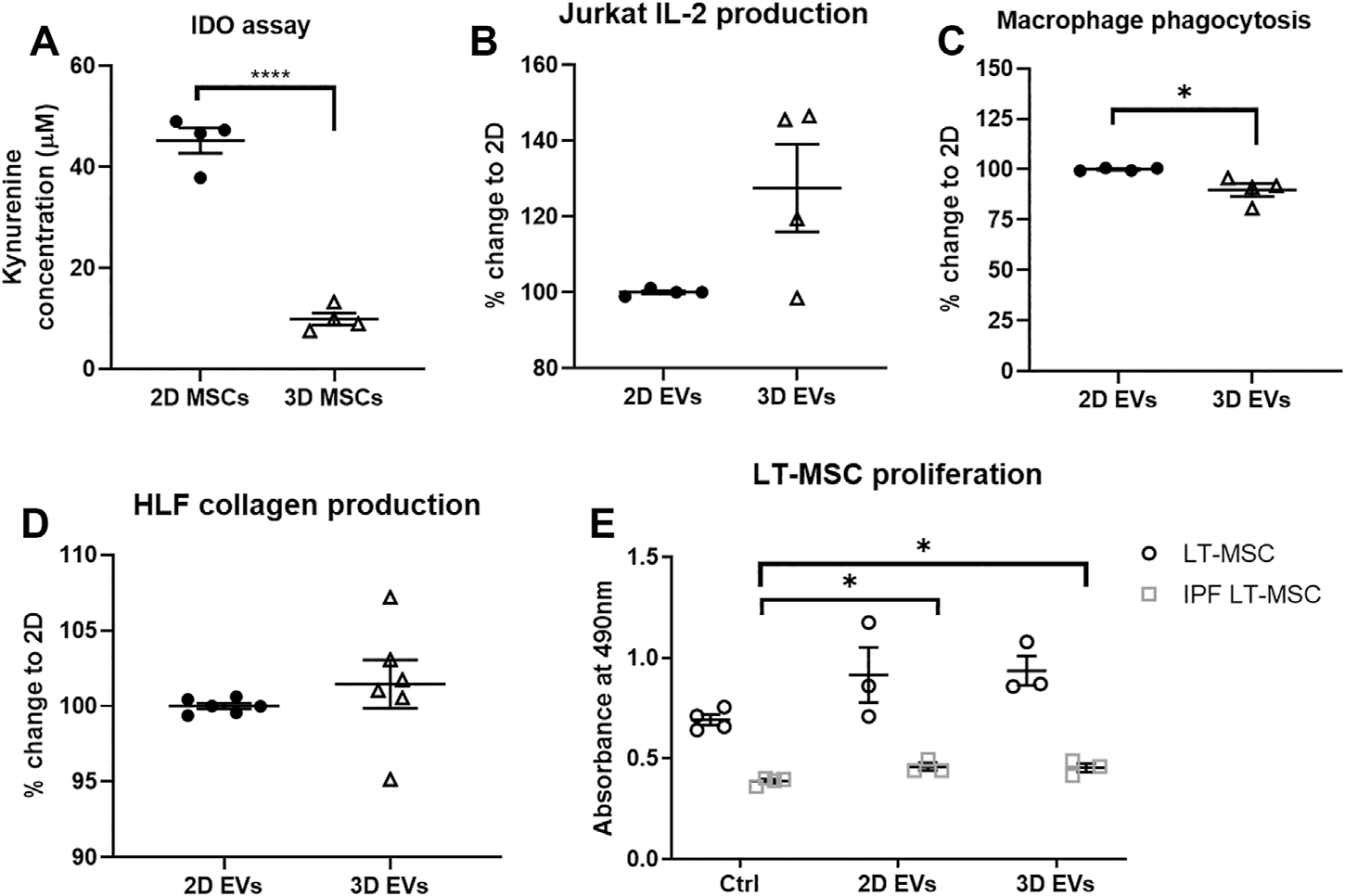
*In vitro* functional assays to test MSC functions following 2D and 3D cultures. **(A)** IDO enzyme activity. **(B)** Changes in IL-2 production by Jurkat cells. **(C)** Changes in macrophage phagocytic activity. **(D)** Changes in collagen deposition upon TGF-β1 activation on human lung fibroblast (HLF) cultures. **(E)** Healthy and IPF LT-MSC proliferation. Unpaired *t*-test used for **(A–D)** and one-way ANOVA followed by Tukey’s post hoc test for multiple comparisons for **(E)**, *p < 0.05, **p < 0.01, ***p < 0.001. All data are shown as mean ± SEM from *n* = 4-6 biological replicate measurements.

The functional effects of EVs derived from 2D and 3D MSC cultures were investigated, with respect to the immunomodulatory, anti-inflammatory and anti-fibrotic effects. To address the immunomodulatory potential, both 2D and 3D MSC cultures were primed for 4 days with 50U/mL IFN-γ and conditioned media were collected. 2D MSCs produced up to 5 times IDO enzyme activity upon IFN-γ stimulation compared to 3D MSCs (Figure 3A, *p* < 0.0001). There were also distinct changes in the 2D and 3D EVs immunomodulatory potencies. First, the effects on IL-2 cytokine production by Jurkat cells were measured and there were no difference in 2D and 3D MSC-EVs (Figure 3B, *p* = 0.0972). The effects of 2D MSC-EVs on macrophage function was further analyzed as macrophages are known to shift between inflammatory (M1) to anti-inflammatory (M2) with increased phagocytic activity. The administration of 3D MSC-EVs resulted in the significant reduction of macrophage phagocytic activity (Figure 3C, *p* < 0.05).

To model the progress of pulmonary fibrosis, human lung fibroblasts (HLFs) were cultured in the presence of 10 ng/ml TGF-β1 for 24 h to induce fibroblast activation, subsequently exposed to 2D and 3D EVs for another 24 h. To test the anti-fibrotic effects of MSC-EVs, 2D and 3D MSC-EVs showed no significant difference in collagen deposition in HLF cultures following inflammation trigger using TGF-β1 (Figure 3D). Further, we observed that lung MSCs from healthy patients proliferate faster than lung MSCs obtained from IPF patients in which there was an approximately 40% reduction in cell metabolic activity. The addition of 2D and 3D MSC-EVs significantly increased cell proliferation on IPF lung MSCs but no overall effect on healthy lung MSCs (Figure 3E).

### Effects of 2D and 3D Extracellular Vesicles on Bodyweight and Lung Function in a Lung Injury Model

**FIGURE 4:**
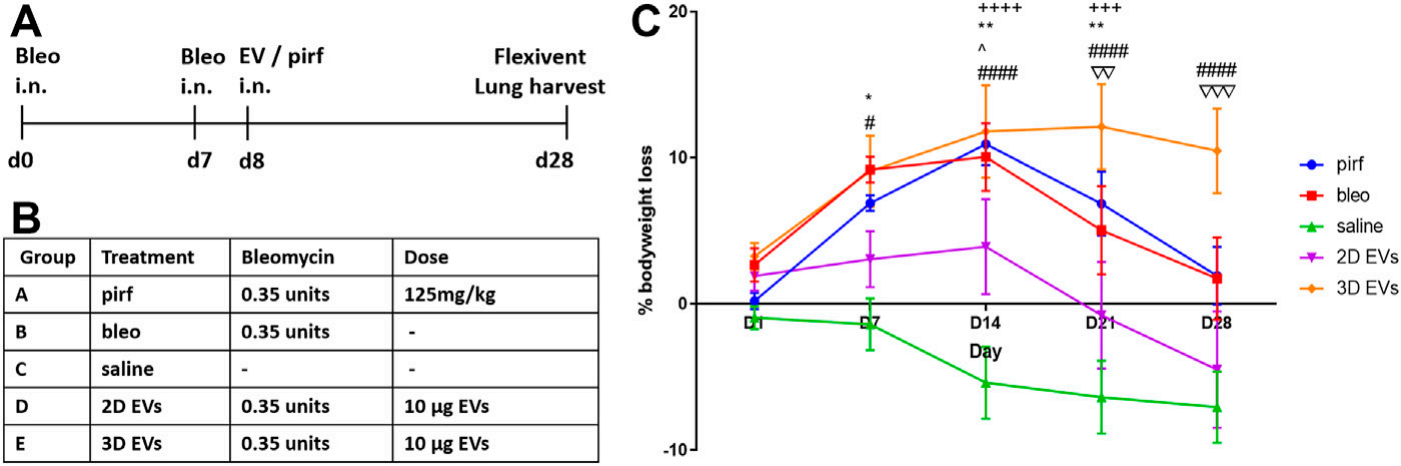
Testing 2D and 3D EVs using an *in vivo* lung fibrosis model with double hit bleomycin in aged mice. **(A)** Timeline for the administration of double hit bleomycin and EV/pirfenidone and endpoint. **(B)** Description of experimental groups and the treatment dose. **(C)** Percentage of bodyweight loss in aged mice throughout the 28 days. Two-way ANOVA was used followed by Tukey’s post hoc test for multiple comparisons, *p < 0.05, **p < 0.01, bleo vs saline, #*p* < 0.05, ####*p* < 0.0001 3D EVs vs. saline, +++ *p* < 0.001, ++++ *p* < 0.0001 pirf vs saline, ^ *p* < 0.05 2D EVs vs saline, ∇∇ *p* < 0.01 2D EVs vs 3D EVs, ∇∇∇ *p* < 0.001 2D EVs vs 3D EVs.

To investigate the therapeutic relevance of our results *in vivo*, a double-dose bleomycin challenge in aged mice was used as a lung injury model that mimics the pathophysiology of IPF. Female C57BL/6 aged mice used in this study had a baseline body weight of 37.3 ± 0.9 g at D0. Bleomycin was administered intranasally to induce injury in mouse lungs at D0 and D7 and the effects of treating the animals at D8 with either 2D or 3D EVs were compared to pirfenidone treatment (current clinical management to reduce IPF progression) (Figure 4A). Details of each experimental group is detailed in Figure 4B. Compared to control mice (saline), animals in pirfenidone group experienced significant bodyweight loss observed at D14 and D21 compared to saline group (Figure 4C). Animals treated with bleomycin-alone followed a similar trend with significant bodyweight loss observed at D7, D14, D21 and a sign of recovery at D28. Bleomycin-treated mice that received 2D EVs showed a less dramatic incline in bodyweight loss with the only significant difference to the saline group only observed at D14 with an increase in body weight was observed at D21 and D28. Animals in 3D EVs group experienced significant bodyweight loss at D7, D14, D21 and D28 days timepoints compared to the saline group.

**FIGURE 5:**
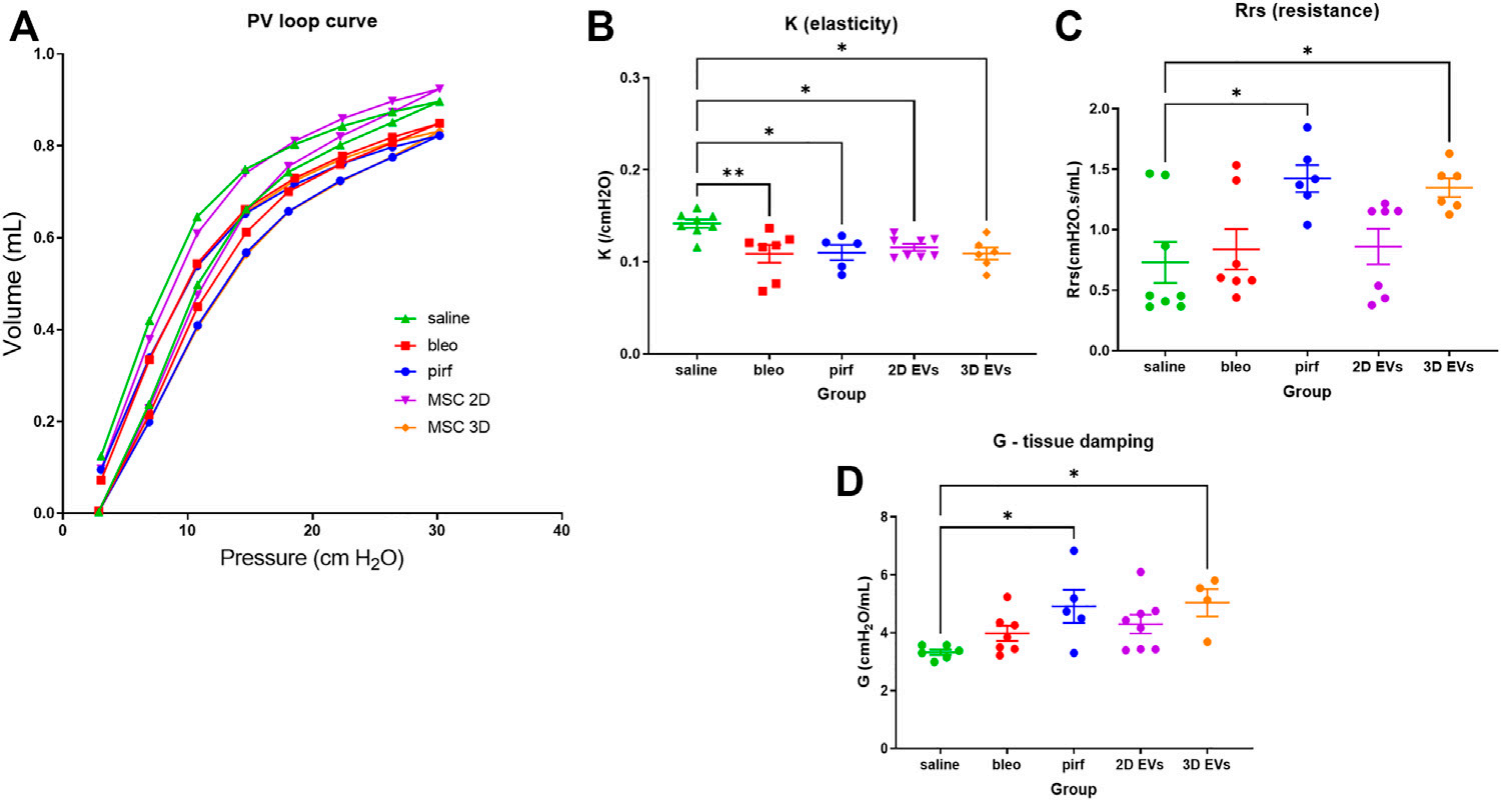
Lung function data in bleomycin-challenged aged mice performed at D28. **(A)** Dynamic PV loop curve of mouse lungs generated from volume-driven PV loops. **(B)** Coefficient of elasticity, K, determined by the Salazar-Knowles equation. **(C)** Dynamic resistance of the respiratory system, Rrs, obtained from single-frequency oscillation technique. **(D)** Lung tissue damping, G, assessed from broadband frequency oscillation technique. All data are shown as mean ± SEM, *n* = 5-8 per experimental group; each symbol represents 1 animal, one-way ANOVA followed by Tukey’s post hoc test for multiple comparisons, *p < 0.05, **p < 0.01.

At day 28, functional testing was performed to determine whether any of the treatments corresponded with improvements in respiratory function using an invasive plethysmography. First, pressure-volume loops (PV) curves were generated by dynamically inflating and deflating the lungs to establish a standard respiratory cycle volume. These PV loops provide information about the way the lungs deform during breathing in healthy and diseased lungs (Phillips et al., 2012; Headley et al., 2018). In the pirf, bleo and 3D EVs groups, there was a downward shift in the PV loops when compared to 2D EVs and saline groups, that becomes more evident once the pressure exceeded 10 cmH_2_O (Figure 5A), indicating stiffer lungs.

The parameter, *K*, which represents the slope of the deflationary limb of the PV loops were fitted to the Salazar-Knowles equation. K was significantly reduced in all groups that received bleomycin compared with the saline group (Figure 5B), which is consistent with the presence of lung fibrosis (Headley et al., 2018). A single-frequency oscillation manoeuvre was then performed using a sinusoidal waveform and the data fitted to the single-compartment model using linear regression to obtain resistance (Rrs). Rrs represents the resistance change in the thorax and it was significantly increased in pirf and 3D EVs groups compared to saline implying a reduction in total respiratory resistance (Figure 5C).

In order to identify changes pertaining to the central and conducting airways as opposed to lung parenchyma, the broadband forced oscillation technique was performed. The broadband forced oscillation technique uses the constant phase model to discriminate between changes in the conducting airways and the lung parenchyma. Lung tissue damping (G), which measures resistance over a broad range of frequencies was increased in a manner similar to Rrs (Figure 5D). No significant difference in G were seen among saline, bleo and 2D EVs groups; however significant differences in G were observed in pirf and 3D EVs compared to saline group.

### Lung Inflammation and Fibroblast Activation

**FIGURE 6:**
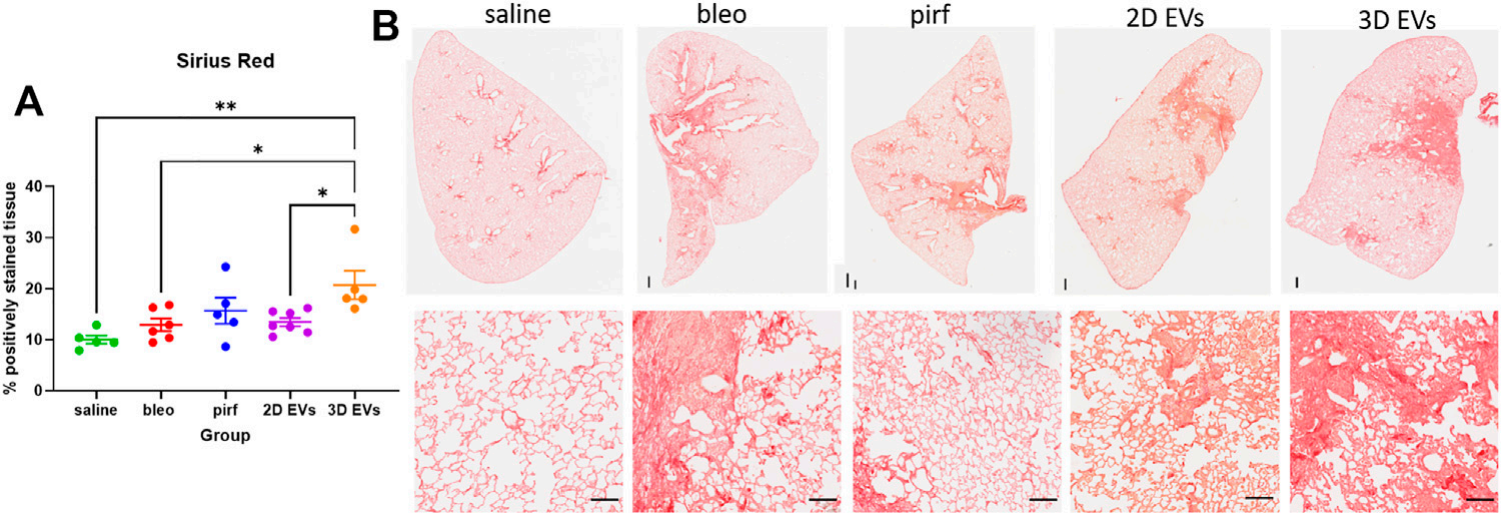
Histological images of lungs at D28 stained with Sirius Red stain and quantification of collagen deposition. **(A)** Percentage of positively stained tissue. **(B)** Representative images of whole lung tissues (upper panel) and lung alveolar area (lower panel). Data represent mean ± SEM, *n* = 5-8 per experimental group; each symbol represents 1 animal, one-way ANOVA followed by Tukey’s post hoc test for multiple comparisons, *p < 0.05, **p < 0.01. Upper panel scale bar is 500 µm and bottom panel scale bar is 100 µm.

As expected, intranasal bleomycin instillation caused a significant inflammatory and fibrotic infiltration in the lung interstitial and intra-alveolar spaces as evidenced by deposition of collagen fibers and obliteration of the alveolar space. Surprisingly, the highest collagen deposition was detected in the lungs of animals treated with the 3D EVs, with significant increase as compared to the saline, bleo, and 2D EVs groups (Figure 6A). There was no difference in collagen deposition between saline and pirf groups suggesting that pirf could attenuate collagen accumulation, the hallmark parameter to define lung fibrosis. Representative images of lung sections from each group demonstrated the extent of collagen accumulation in lung alveolar area with more pronounced Sirius Red staining observed around the airways from 3D EVs group (Figure 6B).

**FIGURE 7:**
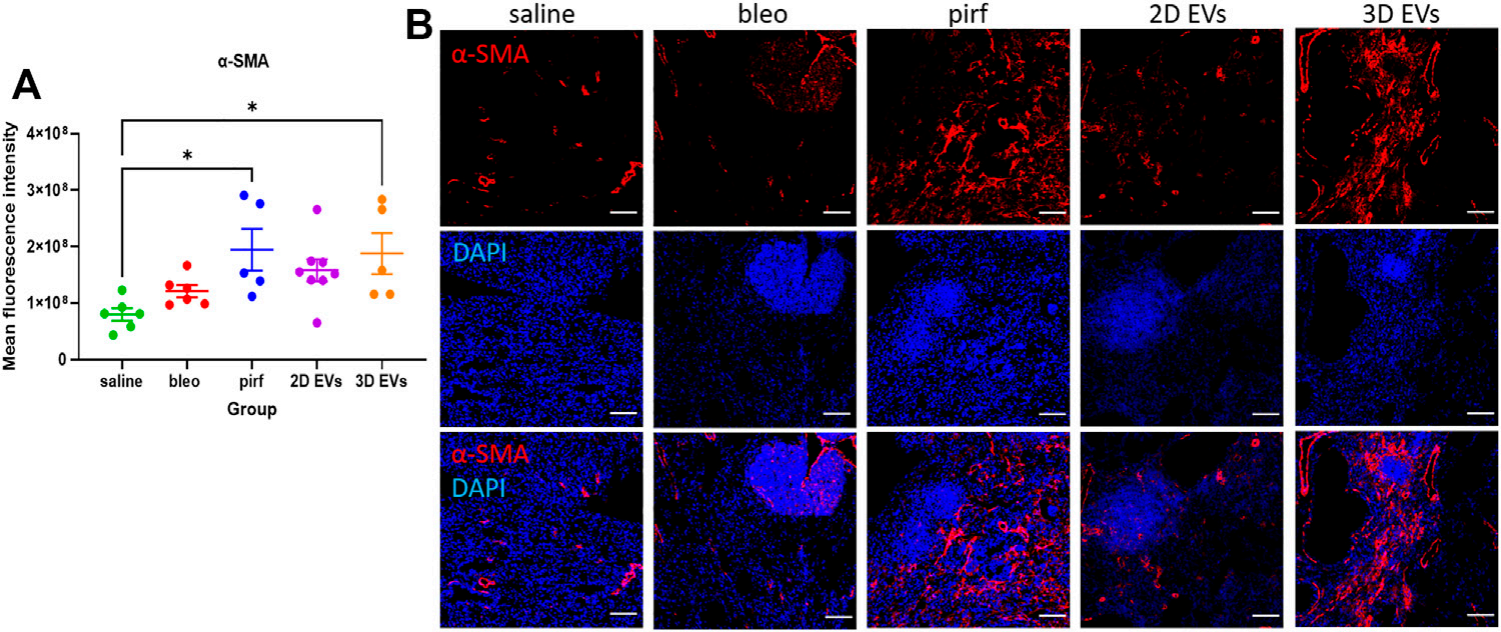
Representative immunofluorescence images of α-SMA (red) staining in lung tissues at D28. **(A)** Mean fluorescence intensity for a-SMA. **(B)** Nuclei staining performed with DAPI (blue). The area with DAPI clusters demonstrated the presence of inflammatory and fibroblastic infiltrate of the lung. Data represent mean ± SEM, *n* = 5-8 per experimental group; each symbol represents 1 animal, one-way ANOVA followed by Tukey’s post hoc test for multiple comparisons, *p < 0.05. Scale bar is 100 µm.

Current evidence suggests that in IPF there is an imbalance in cell signalling which leads to alveolar epithelial senescence and over activation of myofibroblast (Toonkel et al., 2013; El Agha et al., 2017). The anti-fibrotic ability of the treatment groups was determined by quantifying the extent of *α*-SMA staining as a surrogate marker of myofibroblast differentiation. There was no evidence of increased *α*-SMA + cells in bleo and 2D EV groups compared to saline (Figure 7A). However, both pirf and 3D EV groups showed a significant increase in the *α*-SMA expression compared to saline group, indicating a higher prevalence of myofibroblasts in the lung (Figure 7B).

**FIGURE 8:**
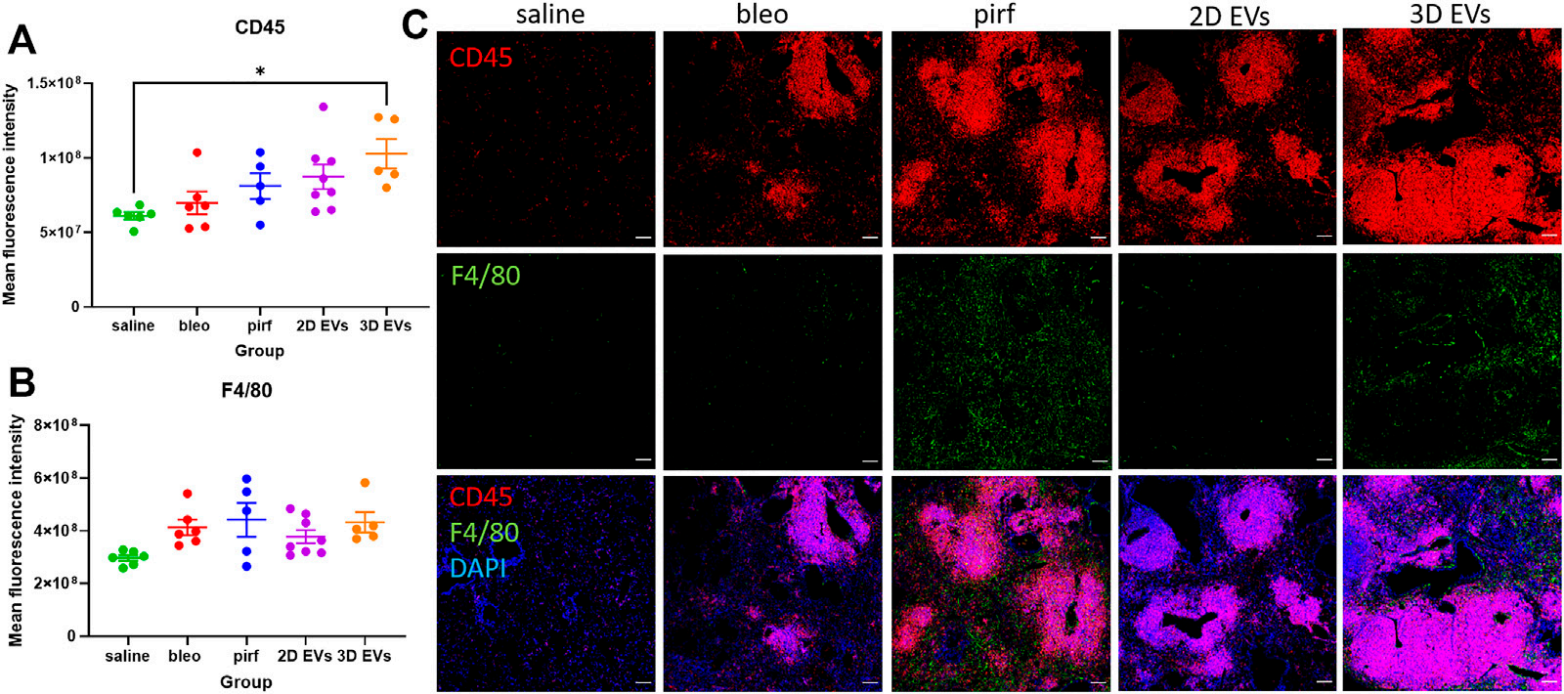
Representative immunofluorescence images of CD45 (red) and F4/80 (green) staining in lung tissues at D28. **(A)** Mean fluorescence intensity for CD45. **(B)** Mean fluorescence intensity for F4/80. **(C)** Nuclei staining performed with DAPI (blue). The area with DAPI clusters demonstrated the presence of inflammatory and fibroblastic infiltrate of the lung. Data represent mean ± SEM, *n* = 5-8 per experimental group; each symbol represents 1 animal, one-way ANOVA followed by Tukey’s post hoc test for multiple comparisons, *p < 0.05. Scale bar is 100 µm.

Inflammatory cell infiltrates were present in the lungs of bleomycin-challenged mice, as shown by the presence of cellular clusters stained with DAPI (Figure 7B). This was then compared to the treatment groups to determine if EV administration would affect leukocyte pulmonary infiltration. Bleo, pirf, and 2D EV groups did not show any significant change to T lymphocytes infiltration, as shown by the quantification of mean fluorescence intensity of CD45 staining (Figure 8A). Interestingly, no change was detected in the number of lung macrophages detected in any of the experimental groups (Figure 8B). These aggregates were primarily composed of T lymphocytes with some lung macrophages (Figure 8C). The data indicate that 3D EVs increased the recruitment of T lymphocytes in lung tissues and resulted in the formation and expansion of these lymphoid aggregates. Further, H&E staining was performed to identify any association between neutrophils infiltration and the inflammatory foci (Supplementary Figure S2) and there was an absence of neutrophils in the experimental groups.

### Influence of Cell Growth Conditions Impact Proteome Composition of Extracellular Vesicles

**FIGURE 9:**
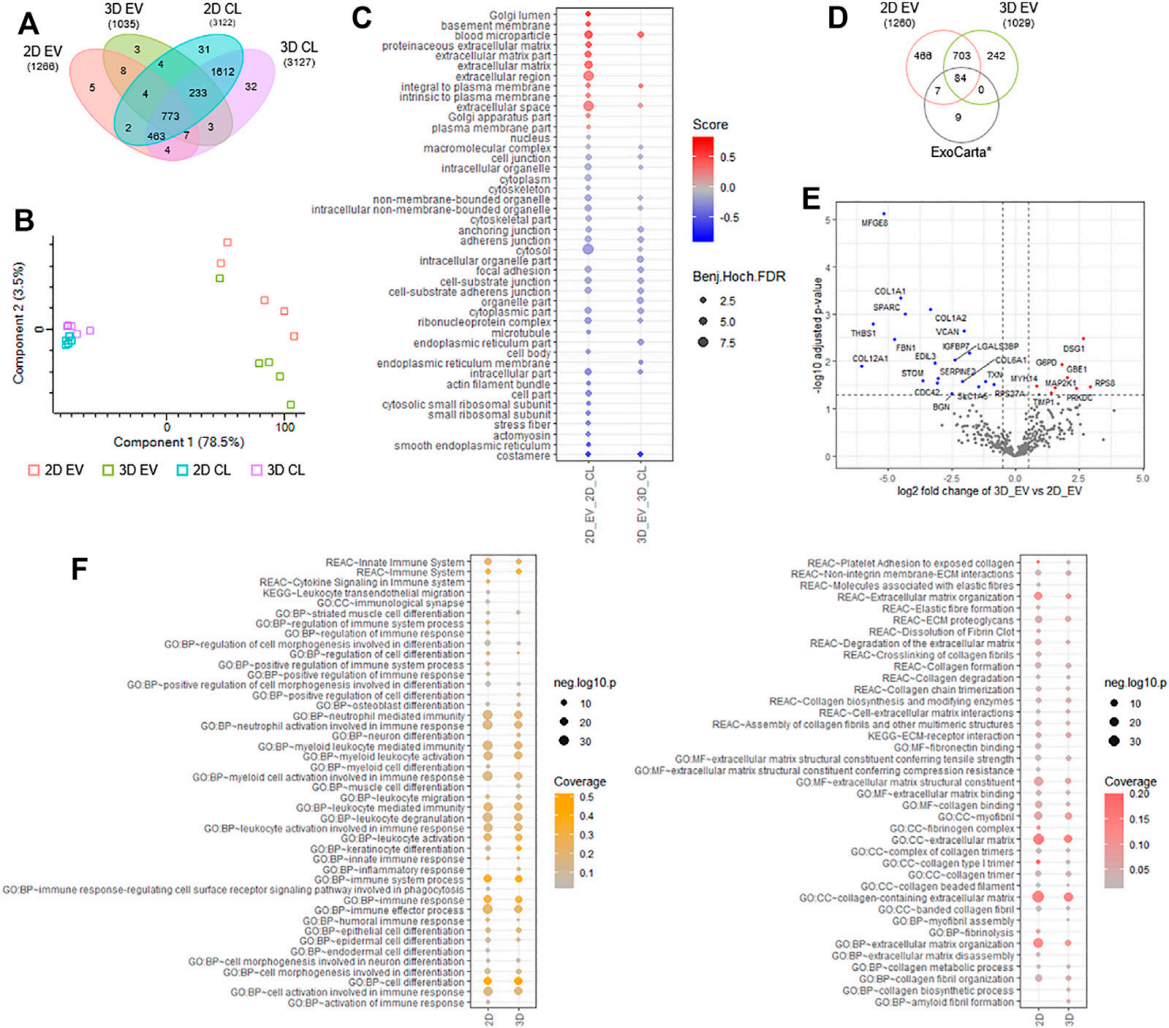
Proteomic analyses of 2D and 3D MSC-derived extracellular vesicles (EVs). **(A)** Venn diagram displaying number of proteins identified in EVs and donor cells grown in 2D and 3D condition using label free-based mass spectrometry from each group. **(B)** principal component analysis of EVs and donor cells grown in 2D and 3D condition. **(C)** Balloon plot displaying Gene Ontology cellular component enrichment terms of EV vs cells in each condition (Benjamin-Hochberg FDR<0.05). **(D)** Venn diagram displaying number of proteins identified in EVs from each group compared to exosome/EV database, ExoCarta (top 100 abundant proteins). **(E)** Volcano plot displaying differential protein expression of EVs derived from 2D and 3D growth condition. **(F)** Balloon plot displaying g:Profiler enrichment terms (Reactome, KEGG pathways, Gene Ontology: biological process, molecular function, cellular component) in EVs from each group (*p* < 0.05). Left panel: “immune-based associated function,” right panel: “fibrosis-related function”.

To gain insight into the proteome composition of EV cargo and whether cell growth conditions in 2D or 3D impacted protein expression, quantitative mass spectrometry was employed (Poh et al., 2021; Zhu et al., 2021). We report for EVs derived from cells cultured in 2D format (2D EVs), 1,266 proteins and from 3D condition (3D EVs), 1,035 proteins (*n* = 5, independent replicates). Distribution of identified proteins in EVs and donor cells is provided in Figure 9A and Supplementary Table S1. We employed quantitative proteomic profiling of EVs based on label-free quantitation and maxLFQ normalization, and differences in proteins identified between each group is shown in Figure 9B and Supplementary Table S2. Highly abundant proteins abundant identified in 2D and 3D EV groups are provided in Supplementary Table S3. We show that in comparison to donor cells, markers of EVs (SDCBP, CD81, CHMP3/5; Supplementary Table S2) and Gene Ontology cellular components including microparticle, plasma membrane, extracellular markers were enriched in EVs, while cytosolic, nuclear, and other organelle markers (e.g. VIM, ACTB, GAPDH) were low in expression in EVs, highlighting the enrichment of EVs in this study (Figure 9C, Supplementary Table S2–S4). Further, we show that proteome composition of EVs from 2D and 3D conditions are distinct from each other and the EV/exosomal database, Exocarta (Figure 9D) (Keerthikumar et al., 2016). Of the 787 proteins common to both EV groups, 466 proteins were unique to 2D EVs, and 242 proteins unique to 3D EVs (Figure 9D).

To further investigate into differential proteome expression of 2D and 3D EVs, we performed stringent inclusion criteria for downstream analyses. Differential expression analysis of 2D and 3D derived EVs revealed 19 specific proteins significantly (*p* < 0.05) upregulated in expression in 2D EVs (e.g., MFGE8, SPARC, VCAN, STOM) and 6 proteins in 3D EVs (e.g., DSG1, GBE1, TIMP1) (Figure 9E, Supplementary Table S5). In addition, we also report 50 proteins unique to 2D EVs including COL8A1 and immunoregulatory components SEC24A, VASP, and complement C1R/S, while 6 proteins were identified unique to 3D EVs, including SOD2 and TFPI2. Overall, we observe 81 proteins differentially expressed/uniquely identified in the EV proteome dependent on the cell culture condition.

Functional enrichment analysis of EV proteome from 2D and 3D group highlighted key processes/functions associated with “immune-based functions” and “fibrosis-related functions” (Figure 9F, Supplementary Tables S6–S7). For immune-based functions, while similarities between 2D and 3D proteome composition in EVs was noted (i.e., immune system, myeloid activation, immune effectors, cell activation, and immune response), several key differences were observed in 3D EVs, including inflammatory response, cell differentiation, in addition to leukocyte migration (Supplementary Table S6). Similarly, for fibrosis-related processes and functions, while similarities between 2D and 3D proteome composition in EVs was observed (i.e., ECM proteoglycan, collagen degradation, ECM-receptor interaction), several key differences were observed in 3D EVs, including collagen synthesis and myofibril assembly (Supplementary Table S7). Further investigation into specific biological processes involved in fibrosis, showed that enrichment for processes such as elastic fibre formation, fibrin clot dissolution, fibronectin binding, fibrinolysis, and ECM disassembly, to be uniquely linked to the contents of 2D EVs.

## Discussion

A significant body of work has demonstrated that 3D spheroid cultures can alter MSC properties, including their secretory profile (Frith et al., 2010; Bartosh et al., 2010; Egger et al., 2018). However, the vast majority of studies investigating derived EVs from MSCs have been performed using MSCs cultured on non-physiological stiff 2D tissue culture plastic substrates. Here, we aimed to investigate how MSC growth as 2D and 3D spheroid cultures could affect the EV production, bioactivity and molecular composition. In agreement with this hypothesis, our results show a remarkable variation between the EVs cargo proteome and therapeutic potencies from 2D and 3D cultured MSCs.

In this study, we used MSCs from bone marrow tissue of healthy donors and established a procedure to obtain the EVs from 3D MSC spheroids cultured in a static system. These were compared to EVs from the MSCs grown in 2D monolayer culture. 3D MSC spheroids could be successfully formed and maintained in a static culture for at least 4 days, a sufficient length of time to collect conditioned-medium. Both 2D and 3D MSC-EVs were characterised with the Minimal Information for Studies of EVs 2018 guidelines (MISEV 2018) (Théry et al., 2019), namely particle size, morphology, surface marker expression, EV-associated protein enrichment compared to parental cells, and low/absent expression of organelle/nuclear components in EVs. Using SP-IRIS technology to characterise EV at a single particle level in terms of particle size, quantity, and surface protein characteristics of EVs, it was shown that both 2D and 3D EVs had a mean particle size of <100 nm, exhibited cup-shaped morphology in TEM, and expressed CD9^+^ CD63^+^ CD81^+^. Together this confirmed that EVs could be isolated from both culture conditions, with further analysis of protein concentration indicating that there were no significant changes to the EV protein yield and concentration between culture conditions. Interestingly, EV production was increased in MSCs cultured in 3D spheroids using poly-(2-hydroxyethyl methacrylate) coating method which produced smaller aggregates (Kim et al., 2018). This could be associated with limited EV release for those cells located deep within the 3D spheroids and will open up future studies to investigate EV secretion and biogenesis in 3D setting. The non-adherent state of cell morphology in smaller 3D aggregates might be important in the enhanced production efficiency of EVs from MSCs.

We next examined the *in vitro* effects of both 2D and 3D EVs with respect to their immunomodulatory, anti-inflammatory and anti-fibrotic potential. Overall these data showed distinct differences in the activity of the 2D and 3D EVs and, surprisingly, the anti-inflammatory and anti-fibrotic effects of the 2D EVs was greater than the 3D EVs. For example, 3D MSC cultures produced significantly less IDO upon IFN-γ stimulation which is an indicator of T cell proliferation. This finding is in line with a recent study that reported aggregation of MSCs also eliminated their T cell suppression ability (Burand et al., 2020). However, treatment of Jurkat cells with both 2D and 3D EVs did not show any significant difference in IL-2 production. Accordingly, treatment of macrophages with 3D EVs resulted in reduced macrophage phagocytosis indicative of a shift in macrophage phenotype into a pro-inflammatory phenotype. In this study, HLF and lung MSCs were used as representative cell populations for *in vitro* analysis because successful lung regeneration is characterised by efficient proliferation and inhibition of lung fibroblast differentiation into myofibroblasts. Activation of HLF with the inflammatory cytokine TGFβ-1 was used to assess whether treatment with EVs could have any impact on collagen production as previously described (Vosdoganes et al., 2013; Tan et al., 2018). In this case, treatment with 2D and 3D EVs did not show any significant change in collagen deposition by HLF. Endogenous lung MSCs have critical role in lung homeostasis such as providing stromal support and promoting tissue repair following injury. Lung MSCs are readily isolated from healthy and IPF patients lung tissue and they have been extensively characterised to confirm their status as bona fide MSCs with high degree of similarity between lung MSCs and bone marrow MSCs (Sinclair et al., 2016a). In healthy humans, lung MSCs reside within the tissue, but in a disease state such as in IPF patients, lung MSCs can differentiate to acquire a profibrotic phenotype. We observed that IPF lung MSCs proliferated at a slower rate compared to healthy lung MSCs. Treatment with 2D and 3D EVs resulted in a significantly increased proliferation in IPF lung MSCs which could help induce tissue repair and regeneration. The secreted factors contained within EVs are likely to synergistically contribute towards lung tissue repair and regeneration by modulating the function of endogenous lung fibroblast and immune cell subsets. Overall, the *in vitro* potency assays suggest that 2D EVs promoted immunosuppression, increased phagocytic activity and anti-fibrotic activity but these potencies were not always replicated in 3D EVs.

*In vivo* double dose bleomycin challenge using aged mice was used in this study to validate the *in vitro* potency assays of 2D and 3D EVs treatment and to mimic an established lung injury. We also included a pirfenidone treatment group which is one of the currently approved drugs for IPF patients and its effectiveness has largely been shown in bleomycin-induced lung injury model with young mice. Assessment of lung mechanics using invasive plethysmography revealed a decrease in elasticity (K) in both pirf and 3D EVs group. This decline was observed in line with the shift in the PV loop curves, and elasticity has been correlated to the severity of lung fibrosis from patient biopsies (Sansores et al., 1996). Both pirfenidone and 3D EVs also showed a downward shift in PV loop curves, increased resistance (Rrs) and increased tissue damping (G). Increased G is consistent with those of a restrictive respiratory disease in which chronic lung injury results in changes in the parenchyma but spares the conducting airways (Headley et al., 2018).

We show that pirfenidone did not completely resolve lung fibrosis but only modestly improved changes to collagen deposition and clearance of immune cell infiltrations. In this regard, the animal model used could be the main differences between this and other studies. Previous studies using younger mice (<12 weeks old) or a single dose of bleomycin where the young mice still possess regenerative potential to repair their lungs therefore the fibrosis can be self-resolved over the time (Peng et al., 2013; Royce et al., 2019). The administration of 2D EVs resulted in faster recovery in bodyweight loss compared to pirfenidone and 3D EVs treatment. We also observed that there was no change in collagen deposition, myofibroblast differentiation, leukocyte and macrophage infiltrations in the lung tissues treated with 2D EVs, indicating that 2D EVs have anti-inflammatory, anti-ECM remodelling, and anti-fibrotic protection against established lung injury. Interestingly, 3D EVs did not show any improvement in most of the aspects of lung fibrosis, demonstrated by a significant increase in bodyweight loss throughout D7-D28, increased in collagen deposition and *α*-SMA + cells (myofibroblast differentiation), as well as an increased presence of leukocyte infiltrations in lung parenchyma.

To better understand the mechanisms that could be driving the observed differences in response to the 2D and 3D EVs, proteomic profiling of EVs was performed using quantitative mass spectrometry. The 2D and 3D culture conditions resulted in different MSC-EVs protein cargo profiles, implying that culture microenvironment leads to non-physiological deviations in MSC biology. In depth proteomic profiling of 2D and 3D MSC-EVs showed that 2D MSC-EVs have an expanded secretion profile and that we observed significant reduction of the number of proteins and their global ontology using functional enrichment analysis in 3D MSC-EVs. Of note and highly relevant to this study in the function of EVs, we report that both 2D and 3D EVs contained key processes involved in cytoskeletal pathways responsible for cell-cell adhesion, ECM organisation, actin depolymerisation and many others. These pathways are consistent with the changes between 2D and 3D culture microenvironment–primarily with proteome differences associated with immune-based associated function and fibrosis-related annotations. Importantly, we report specific EV cargoes (242 proteins in EVs from 3D culture) unique to this subset that are not detectable in EVs secreted by cells cultured in 2D conditions. This is also supported by several unique functional enrichment categories in 3D EVs, including inflammatory response, cell differentiation, in addition to leukocyte migration.

It is clear that both parental cell identity and culture parameters impact upon EV cargo and function. With respect to 2D and 3D culture conditions, these have profound effects on the cellular phenotype and cytoskeletal organisation. One evident change upon spheroid formation is the development of a hypoxic environment in the core of each spheroid and this will vary depending on the spheroids size (Petrenko et al., 2017). Also, the alteration in mechanophysical properties might be such a significant effect in MSC spheroids and these have been shown to cause drastic change in the gene expression profile, epigenetic modifications, as well as stem cell potencies (Guo et al., 2014).

Another aspect through which this relates to EVs is that cytoskeletal components play important roles in the secretion of EVs, although the machineries involved in the release of EVs are still at an early stage of comprehension. Budding and release of EVs from cells require altered actin polymerisation under the plasma membrane, followed by contraction of associated molecular motors (kinesins and myosins), molecular switches (small GTPases) and other fusion machineries (Raposo and Stoorvogel 2013). Changes in MSC cell morphology following cellular aggregation/spheroids formation are therefore likely to affect the expression of cytoskeletal network proteins and also the subsequent cargo sorting in EVs. The contraction of actin microfilaments and/or actin polymerisation is necessary to facilitate the release of EVs. 3D culture has been shown to reduce MSC cell size and also increase the number of membrane-bound vesicles on the cell surface. 3D MSCs reduced the cell volume by up to 70% and this has been associated with decreased entrapment of MSCs in the lungs following intravenous infusion and a markedly increased levels of circulating MSCs (Wang et al., 2014). The level of actin polymerisation was observed to be lower in 3D MSC cultures and therefore lowering actin cytoskeleton tension may facilitate vesicle excretion (Mo et al., 2017; Kim et al., 2018). Impact of mechanical cues demand further investigation in modulating MSC fate in different culture microenvironment and the downstream impact in MSC paracrine outputs such as EV release and functions. The possible contribution of all these different cytoskeletal and cell-cell junction components in mediating EV secretion in MSC spheroids has not been explored.

The differences in paracrine signalling and function mediated by EVs from 2D and 3D MSCs could be the consequence of extensive cell-cell interactions within the 3D spheroids which could subsequently impact the EV biogenesis and release, therefore impacting the regenerative function of MSC-EVs. In our hands, 3D EVs resulted in suppression of immunomodulatory activity, pro-inflammatory, and pro-fibrotic environment.

## Concluding Remarks and Future Directions

This study adds a new dimension to the current understanding of MSC secretome biology and highlights that the microenvironmental conditions will affect EV content and function. Information from this study will underpin future screening of appropriate microenvironmental cues in order to produce EVs with high potencies and therapeutic potential. Paracrine mechanisms allow MSCs to orchestrate a regenerative response by instructing desirable functions in endogenous cell populations. The ability of MSCs to accomplish this effect can be modulated using microenvironmental cues is an exciting avenue to improve the outcomes of MSC-based therapeutics. The work reported here provides new insight that EVs obtained from aggregation of MSCs into 3D spheroids did not provide the necessary bio-instructive cues to maintain and direct their therapeutic potential. EVs isolated from static 3D MSC cultures are not a viable alternative to 2D monolayer MSC as 3D EVs did not retain MSC anti-fibrotic and anti-immunomodulatory properties both *in vitro* and *in vivo*. Morphological changes and compacted cell sizes are known features in MSC spheroids and potentially offer some advantages for scale up EV manufacturing that aim to produce highly potent EVs for a therapeutic application. Future studies will focus on exploring several avenues in dynamic 3D culture conditions and modulating spheroid sizes as the next steps towards bringing scalable MSC-EVs manufacturing for clinical applications.

The outcome highlights critical differences in EVs obtained from different culture microenvironment, which should be considered when scaling up MSC culture for clinical manufacturing. The field is yet to combine all of these microenvironmental factors to produce an optimised platform for MSC expansion and EV purification. It is imperative that the platform for EV production is not only more efficient than current standard protocols, but that also produces a higher quality and potent product and that is more cost effective. There are substantial efforts worldwide attempting to address these challenges, with progress in both our fundamental understanding of MSC biology, cell-substrate interactions, and microenvironmental impacts of cell behaviour on downstream EV production will rapidly pushing the field towards sensible and cost-effective solutions.

## Data Availability

Mass spectrometry RAW and processed data have been deposited to the ProteomeXchange Consortium *via* the PRIDE partner repository and are available *via* ProteomeXchange with identifier PXD029766.
