# Supplementary material for: Effect of 2D and 3D Culture Microenvironments on Mesenchymal Stem Cell-Derived Extracellular Vesicles Potencies
Source: Front Cell Dev Biol. 2022 Feb 14;10:819726. doi: 10.3389/fcell.2022.819726 (PMC8882622; doi:10.3389/fcell.2022.819726)
Supplement: Supplementary file 2 [file DataSheet1.docx]

**Supplementary Figures**


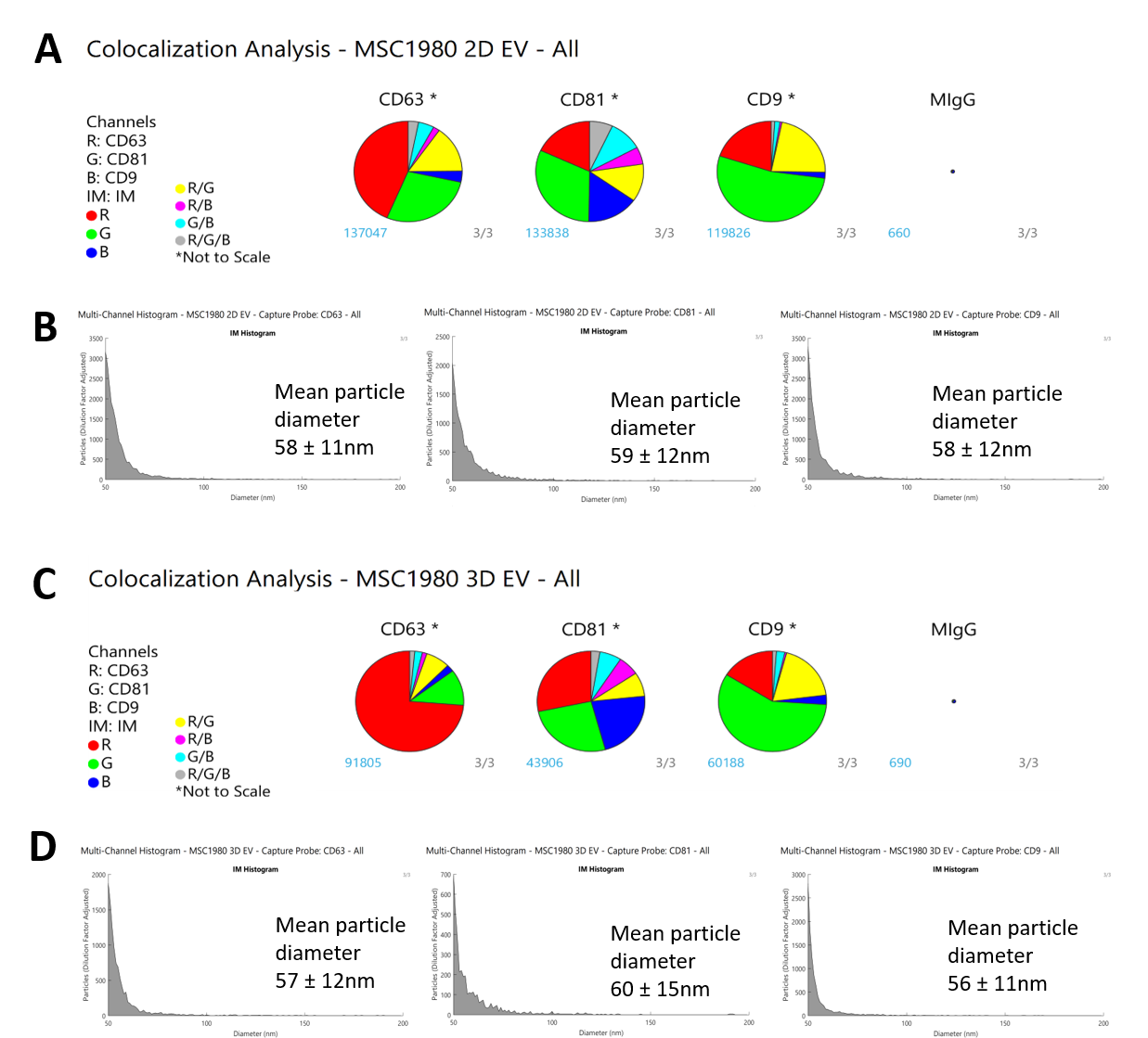


**Supplementary Figure 1. Representative EV profiles from 2D and 3D culture obtained from single particle interferometric reflectance imaging sensing (SP-IRIS) analysis.** (A, C) Distribution of single/double/triple positive particles detected in each capture spot. (B, D) Particle size quantification in each captured antibody, CD63, CD81, and CD9.


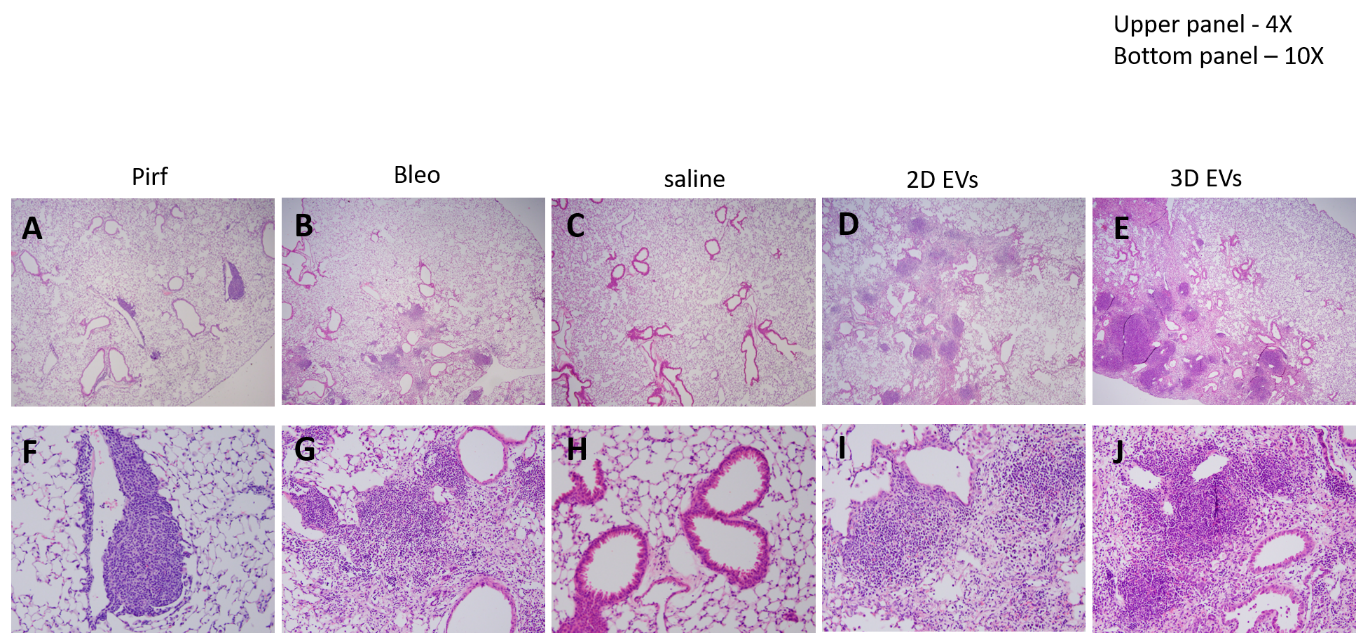


**Supplementary Figure 2. H&E staining on lung tissues within the inflammatory foci.** Upper panel are 40X magnification images and bottom panel are 100X magnification images.
